# Retrospective Evaluation of Hematological Parameters in COVID-19 Patients: Insights From the Emergency Department

**DOI:** 10.7759/cureus.61258

**Published:** 2024-05-28

**Authors:** Ahmed Jerah

**Affiliations:** 1 College of Applied Medical Sciences, Jazan University, Jazan, SAU

**Keywords:** survival outcomes, cox regression analysis, neutrophil percentage, covid-19 outcomes, hematological parameters

## Abstract

Background: This retrospective study evaluated hematological parameters in coronavirus disease 2019 (COVID-19) patients to gain clinical insights.

Methods: Data from the Emergency Department of Samtah General Hospital, Samtah, Saudi Arabia, were analyzed, focusing on the parameters measured during hospital admission. This study was conducted between April 2020 and October 2021. Associations between hematological parameters and COVID-19 outcomes were examined in 153 participants, including 23 deceased individuals.

Results: The chi-square test results indicated no significant associations (P >0.05) between sex, body mass index (BMI), age, and disease outcome in the study population. However, a significant association was observed between neutrophil percentage and disease outcome, whereas no significant associations were found for red blood cell count, hemoglobin level, monocyte percentage, eosinophil percentage, and basophil percentage. Cox regression analysis revealed a significant association between neutrophil count (considered a categorical covariate) and survival outcomes (P = 0.030). However, specific neutrophil categories (50-70 and >70) were not significantly associated with survival.

Conclusions: Integrating hematological parameters into COVID-19 clinical guidelines and decision-support tools holds promise for enhancing patient care and outcomes.

## Introduction

The coronavirus disease 2019 (COVID-19) pandemic, caused by the novel severe acute respiratory syndrome coronavirus 2 (SARS-CoV-2), continues to pose significant challenges to the global healthcare system [1]. Since its emergence in late 2019, the virus has rapidly spread globally, leading to millions of infections and a considerable number of fatalities [2]. The severity and clinical manifestations of COVID-19 vary greatly among individuals, with some experiencing mild flu-like symptoms and others developing severe respiratory distress and multiorgan dysfunction [3]. Identification and understanding of key prognostic indicators and biomarkers are crucial for effective management and risk stratification in COVID-19 patients [2,4].

Hematological parameters encompassing various blood components are essential indicators of overall health status [5]. They have been extensively studied in different disease conditions and can provide valuable insights into disease pathogenesis, severity, and prognosis. In the context of COVID-19, emerging evidence suggests that hematological abnormalities are frequently observed in affected individuals [6,7]. The hematological abnormalities observed in COVID-19 patients can vary and may include changes in white blood cell counts, red blood cell indices, platelet counts, and coagulation parameters. These alterations reflect the complex interplay between the virus, immune responses, and physiological processes in the body [8]. Studying these hematological parameters can aid in understanding the underlying mechanisms of COVID-19 and its impact on various organ systems [9].

The emergency department (ED) plays a critical role in the initial assessment and triage of COVID-19 patients [10]. Serving as the primary point of contact for individuals seeking urgent medical care, it acts as a crucial gateway to the appropriate management and allocation of healthcare resources. Prompt and accurate evaluation of hematological parameters in COVID-19 patients in the ED can assist healthcare professionals in identifying individuals with severe disease manifestations who require immediate intervention and specialized care [11,12]. By assessing hematological parameters in COVID-19 patients in the ED, healthcare providers can gain valuable insights into disease severity and progression [13]. Abnormalities in these parameters may serve as early warning signs of potential complications such as cytokine storm, coagulopathy, or acute respiratory distress syndrome (ARDS). Identifying these markers promptly allows for timely interventions, such as initiating antiviral treatments, providing supplemental oxygen, or facilitating early referral to intensive care units [14]. Moreover, the evaluation of hematological parameters in COVID-19 patients in the ED can contribute to the efficient allocation of healthcare resources [14]. By identifying individuals with severe disease presentations, healthcare providers can prioritize the allocation of limited resources, including hospital beds, intensive care units, and medical equipment, to those who urgently need them. This strategic resource allocation helps optimize patient outcomes and enhances the overall capacity of the healthcare system to manage the pandemic effectively [2,5,6,8,11].

The current study aimed to present a comprehensive retrospective analysis of hematological parameters in COVID-19 patients, focusing on insights obtained from the ED. By examining a large cohort of patients and analyzing their hematological profiles, we sought to shed light on the potential clinical significance of these parameters in the context of COVID-19. By analyzing the data, we aimed to determine whether specific hematological parameters exhibited significant differences between mild, moderate, and severe COVID-19 cases. Furthermore, the potential association between hematological abnormalities and clinical outcomes was investigated, including disease progression, requirement for intensive care, and mortality. A subgroup analysis based on age, comorbidities, and other relevant factors was performed to identify potential risk factors and aid in risk stratification.

## Materials and methods

Population and study site

In this retrospective analysis, a total of 153 patients (82 males and 71 females) with a median age of 54.73 years were included. The study was conducted at Samtah General Hospital, Samtah, Jazan, Saudi Arabia, between April 2020 and October 2021. Demographic information and biochemical parameters of the patients were retrieved from electronic medical records. The study was approved by the Ethical Committee of the Health Directorate in the Jazan Region (approval number: 2364) and strictly followed the ethical guidelines outlined in the Declaration of Helsinki. As a retrospective study, there was no direct contact with patients, ensuring strict adherence to privacy and confidentiality guidelines. The diagnosis of COVID-19 was confirmed using reverse transcriptase-polymerase chain reaction (RT-PCR) analysis of nasopharyngeal swab specimens following the guidelines established by the Centers for Disease Control and Prevention in Saudi Arabia [15].

Inclusion and exclusion criteria

Initially, a total of 2010 individuals who tested positive for COVID-19 and underwent screening were considered for inclusion in the study. The inclusion criteria were laboratory-confirmed cases of COVID-19 and the availability of complete hematological profiles upon admission. Patients with preexisting hematological disorders or incomplete medical records were excluded from the analysis. Electronic health records provided a wealth of information, including demographic data, clinical presentation, laboratory results, disease severity, and outcomes. Following the application of these inclusion criteria, a final cohort of 153 patients met all necessary requirements and were included in the study.

Measures

The retrospective analysis included variables such as sex, age, weight, height, body mass index (BMI), mortality, and hematological indices. The primary objective of this study was to evaluate the hematological parameters of patients with COVID-19 and identify any significant associations between these parameters and disease outcomes.

Data analysis

The data were analyzed using IBM SPSS Statistics for Windows, Version 20.0 (Released 2011; IBM Corp., Armonk, New York, United States) through statistical description and analysis. Continuous variables are presented as medians or simple ranges, depending on their distribution. Categorical variables, on the other hand, were summarized using counts and percentages. The normality of the distribution for continuous variables was assessed using the Kolmogorov-Smirnov test. The variables were found normally distributed (P>0.05). A one-sample t-test was employed to compare the levels of biochemical parameters (mean ± SD) with their respective reference intervals. Statistical significance was set at a threshold of P < 0.05.

## Results

Table 1 presents the distribution of variables and disease outcomes, along with the results of the chi-squared test to assess the association between these variables and disease outcomes. The variables analyzed in this study included sex, BMI, comorbidities, and age. Among the males, 82 (53.6%) were alive and 73 (47.7%) died. Among the females, 71 (46.6%) were alive and 57 (37.3%) died. The Chi-squared test yielded a test statistic value of 2.7 (p-value = 0.131), indicating that there was no significant association between sex and disease outcome. BMI was categorized into four groups: underweight, normal-weight, overweight, and obese; among participants classified as underweight, 13 (8.7%) were alive and one (0.7%) died. The chi-square test resulted in a test statistic value of 4.01 (p-value = 0.26), suggesting no significant association between BMI and disease outcome. The participants were divided into three age groups: less than 40 years, 40-60 years, and > 60 years; among participants aged less than 40 years, 41 (26.8%) were alive and five (3.3%) were deceased. The chi-square test yielded a test statistic value of 0.59 (p-value of 0.74), indicating no significant association between age and disease outcomes. In summary, based on the results of the chi-squared test, there was no significant association between sex, comorbidities, BMI, age, and disease outcome in the study population.

**Table 1:** Sex, BMI, and age and their distribution among the categories of disease outcome (alive/dead) (N= 153) Chi-square test was used to analyze the association

Variables		Disease outcome		Chi-squared test
	Total participants, n (%)	Alive, n (%)	Dead, n (%)	Test statistics (value)
Gender				
Male	82 (53.6)	73 (47.7)	9 (5.9)	2.7 (0.131)
Female	71 (46.6)	57 (37.3)	14 (9.2)	
Body Mass Index (kg/m^2^)				
Underweight (<18.5)	13 (8.7)	12 (8.0)	1 (0.7)	4.01 (0.26)
Normal (18.5-24.9)	54 (36.0)	47 (31.3)	7 (4.7)	
Overweight (25-29.9)	58 (38.7)	50 (33.3)	8 (5.3)	
Obese (>30)	25 (16.7)	18 (12.0)	7 (4.7)	
Age (years)				
<40	41 (26.8)	36 (23.5)	5 (3.3)	0.59 (0.74)
40-60	30 (19.6)	26 (17.0)	4 (2.6)	
>60	82 (53.6)	68 (44.4)	14 (19.1)	
Comorbidities				
No	62 (40.5)	51 (33.3)	11 (7.2)	0.60 (0.44)
Yes	91 (59.5)	79 (51.6)	12 (7.8)	
Total	153 (100)	130 (85.0)	23 (15.0)	

The association between various hematological parameters and disease outcomes was examined. Significant associations were found for the neutrophil percentage (χ² = 8.5, p = 0.014). However, no significant associations were observed for red blood cell count (RBC) (χ² = 0.48, p = 0.79), hemoglobin level (Hb) (χ² = 0.04, p = 0.85), monocyte percentage (χ² = 2.25, p = 0.32), eosinophil percentage (χ² = 1.11, p = 0.29), and basophil percentage (χ² = 0.7, p = 0.79) (Table 2).

**Table 2:** Hematological parameters and their distribution among the categories of disease outcome (alive/dead) (N= 153). Chi-squared test was used to analyze this association

Variables		Disease outcome		Chi-squared test
	Total participamts, n (%)	Alive, n (%)	Dead, n (%)	
WBC (%)				
<4.8	33 (21.6)	25 (16.3)	8 (5.2)	3.1 (0.21)
4.8–10.8	73 (47.7)	65 (42.5)	8 (5.2)	
>10.8	47 (30.7)	40 (26.1)	7 (4.6)	
RBC (mcL)				
<4.1	37 (24.2)	32 (20.9)	5 (3.3)	0.48 (0.79)
4.1-6.0	105 (68.6)	88 (57.5)	17 (11.1)	
>6.0	11 (7.2)	10 (6.5)	1 (0.1)	
Hemoglobin (g/L)				
>12	76 (49.7)	65 (42.5)	11 (7.2)	0.04 (0.85)
<12	77 (50.3)	65 (42.5)	12 (7.8)	
Neutrophil (%)				
<50	24 (15.8)	23 (15.1)	1 (0.7)	8.5 (0.014)
50-70	48 (31.6)	35 (23.0)	13 (8.6)	
>70	80 (52.6)	71 (46.7)	9 (5.9)	
Lymphocytes (%)				
<20	83 (54.6)	73 (48)	10 (6.6)	5.37 (0.068)
20-40	44 (28.9)	44 (28.9)	13 (8.6)	
>40	12 (7.9)	12 (7.9)	0 (0)	
Monocytes (%)				
<2	9 (5.9)	8 (5.3)	1 (0.7)	2.25 (0.32)
2-9	97 (63.8)	85 (55.9)	12 (7.9)	
>9	46 (30.3)	36 (23.7)	10 (6.6)	
Eosinophil (%)				
0-5	146 (96.1)	123 (80.9)	23 (15.1)	1.11 (0.29)
>5	6 (3.9)	6 (3.9)	0 (0.0)	
Basophil (%)				
0.0-0.2	102 (67.1)	86 (56.6)	16 (10.5)	0.7 (0.79)
>0.2	50 (32.9)	43 (28.3)	7 (4.6)	
Total	153 (100)	130 (85)	23 (15)	

Table 3 summarizes the results of the statistical tests that compared the average values of various biochemical parameters to their normal ranges. The parameters analyzed included WBCs, RBCs, neutrophils, lymphocytes, monocytes, eosinophils, and basophils. The findings indicated significant deviations from the normal range for WBCs, RBCs, neutrophils, monocytes, and basophils. These parameters showed significant mean differences and p-values below the chosen significance level (p < 0.05), suggesting significant differences from the normal range. However, lymphocyte and eosinophil percentages did not exhibit significant deviations, as their p-values were above the significance level. These parameters showed no significant mean differences from normal ranges. These results provide valuable information regarding the extent of deviation for each parameter and help identify potential abnormalities in the studied population.

**Table 3:** Comparison of the average of each biochemical parameter to its normal range *One-Sample Test. The blood analysis encompassed several parameters

Parameters	Mean difference	95% Confidence intervals		p-value*
		Lower	Upper	
White Blood Cells (mcL)	8.00	4.41	11.61	0.000
Red Blood Cells (mcL)	0.575	0.19	0.95	0.007
Neutrophils (%)	12.73	11.14	14.33	0.000
Lymphocytes (%)	-10.95	-32.73	10.82	0.303
Monocytes(%)	4.37	2.15	6.58	0.000
Eosinophils (%)	6.67	-4.08	17.41	0.172
Basophils (%)	0.36	0.22	0.50	0.000

**Figure 1:**
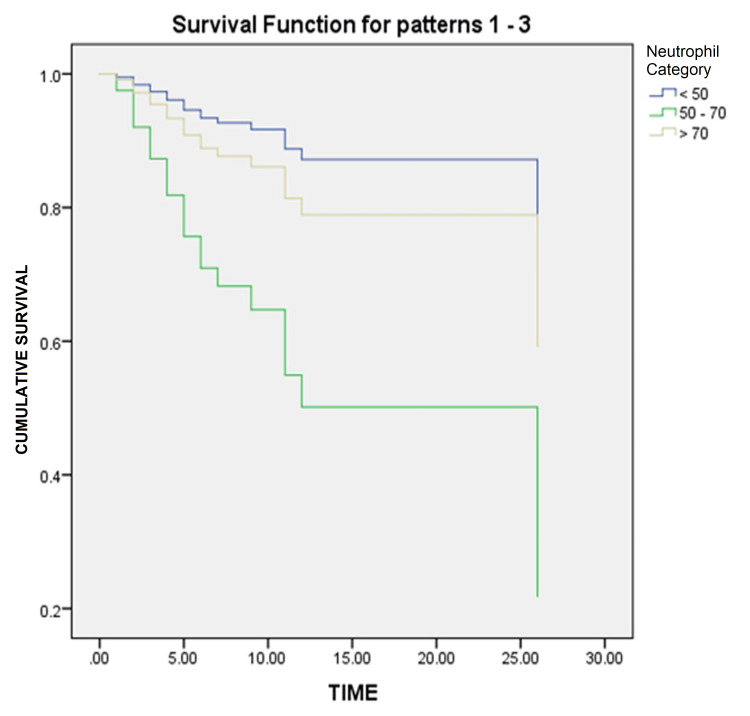
Survival curve

Table 4 and Figure 1 present the results of Cox regression analysis examining the impact of neutrophils, considered as a categorical covariate, on survival outcomes. The analysis revealed that the neutrophil count was significantly associated with survival, as indicated by a p-value of 0.030. The reference group (<50 neutrophils) served as the baseline for comparison. Individuals in the neutrophil 50-70 category showed no statistically significant association with survival (p = 0.120), with an OR of 5.031 (95%CI: 0.657-38.529). Those in the neutrophil > 70 category also exhibited no significant association (p = 0.606), with an OR of 1.729 (95%CI: 0.215-13.875).

**Table 4:** Cox regression for the analysis of survival. Neutrohil was considered as used in the model as categorical covariate.

Covariate	p-value	Odds ratio	95.0% Confidence intervals	
Neutrophils (%)	0.030		Lower	Upper
<50 (Reference group)				
50-70	0.120	5.031	0.657	38.529
>70	0.606	1.729	0.215	13.875

It is important to interpret these results with caution considering the wide CIs and potential limitations of the study. Consulting with a statistician or researcher familiar with the data is advised for a more comprehensive understanding of Cox regression findings. 

## Discussion

The objective of this study was to retrospectively evaluate hematological parameters in COVID-19 patients, specifically focusing on insights gained from the ED. By retrospectively evaluating these hematological parameters, the researchers aimed to gain a better understanding of how COVID-19 affects the blood profile of patients presenting to the ED. This analysis provides valuable insights into the hematological manifestations and potential abnormalities associated with COVID-19.

Evaluating hematological markers in COVID-19 patients upon their arrival at the ED is of utmost importance for diagnosis, prognosis, and management purposes. These markers provide valuable insights into the blood profile, enabling early detection of complications like thrombosis and immune dysregulation. They also aid in assessing disease severity and guiding appropriate care. Certain markers such as D-dimer and C-reactive protein (CRP) have prognostic implications, indicating a higher risk of poor outcomes and mortality. Utilizing these markers, tailored treatment strategies can be developed such as considering anticoagulant therapy for patients with elevated D-dimer levels [6,10,16]. Studies conducted by Zhou et al. [17] and Henry et al. [18] have underscored the association between hematological markers and COVID-19 outcomes. Ongoing research in this field is expected to further enhance our understanding and improve patient management.

This study examined the association between various hematological parameters and disease outcomes. Significant associations were found for the neutrophil percentage (χ² = 8.5, p = 0.014). Considerable evidence has been gathered since the onset of the COVID-19 pandemic, highlighting the significant role of neutrophils in the underlying disease processes, particularly among individuals experiencing severe clinical courses [19]. Previously viewed as a relatively uniform cell type, recent investigations into neutrophils have revealed their intriguing diversity in terms of gene expression patterns and functional capabilities, shedding light on their developmental trajectories [19,20]. These novel discoveries hold great importance in comprehending the multifaceted involvement of neutrophils, not only in COVID-19 but also in various other acute or chronic inflammatory conditions, whether communicable or non-communicable [21,22]. Abnormal neutrophil levels in COVID-19 patients are believed to be influenced by a dysregulated immune response triggered by SARS-CoV-2. The release of pro-inflammatory cytokines, often referred to as cytokine storm, can lead to the recruitment and activation of neutrophils, contributing to the overall inflammatory response and tissue damage seen in severe cases of COVID-19 [18,23].

The Cox regression analysis presented in Table 4 and Figure 1 examined the impact of neutrophils, treated as a categorical covariate, on survival outcomes. In the context of the information provided, Cox regression analysis was used to evaluate the impact of neutrophil levels (categorized as <50, 50-70, and >70) on survival outcomes. The analysis yielded a p-value of 0.030, indicating a statistically significant association between the neutrophil count and survival. This suggests that neutrophil levels affect the likelihood of survival. It is crucial to interpret these results cautiously considering the wide confidence intervals and potential limitations of the study. Zhou et al. conducted a study in Wuhan, China to identify risk factors associated with ARDS and death in COVID-19 patients [17]. They employed survival analysis techniques and identified various factors that increased the risk of adverse outcomes. Another study by Wu et al. focused on the clinical course and risk factors of mortality in adult COVID-19 patients in Wuhan, China. Survival analysis was used to assess the factors associated with patient outcomes, providing insights into disease progression and potential predictors of mortality [24]. Barry et al. examined the clinical characteristics and outcomes of hospitalized COVID-19 patients in an area with previous Middle East Respiratory Syndrome (MERS) outbreaks [25]. They employed survival analysis to identify the factors that may influence disease outcomes in this specific population. Mikami et al. conducted a study in New York City to evaluate the risk factors of mortality in COVID-19 patients [26]. They used survival analysis techniques to identify factors associated with an increased likelihood of death, providing valuable information for risk stratification and clinical management. Arentz et al. investigated the characteristics and outcomes of critically ill COVID-19 patients in Washington State [16]. They employed survival analysis to examine the factors associated with poor outcomes in this specific patient population. These studies utilized survival analysis techniques, such as Cox regression, to analyze various factors and their impact on survival outcomes, mortality risk, and disease progression in COVID-19 patients. The findings of these studies contribute to our understanding of the disease and help identify potential risk factors for adverse outcomes in COVID-19 patients.

Recommendations

Prospective studies, including cohort and randomized controlled trials, should be conducted to establish causal relationships, address confounding factors, and capture the temporal dynamics of hematological changes. Standardized protocols for measuring and monitoring hematological parameters should be developed to enhance consistency and comparability across studies. Identifying specific hematological parameters that are strongly associated with adverse outcomes can aid in early identification and risk stratification. Personalized treatment approaches based on individual hematological profiles may improve patient outcomes. Long-term follow-up studies can assess the effects of COVID-19 on hematological parameters. Collaboration, data sharing, and large-scale multicenter studies can overcome these limitations and provide comprehensive insights. Integrating findings into clinical guidelines and decision-support tools can assist healthcare professionals in risk assessment, treatment planning, and monitoring strategies. These recommendations should be considered in the context of the findings and limitations of a specific study.

Limitations and strengths

This study may have encountered several limitations associated with retrospective studies examining hematological parameters in COVID-19 patients. These limitations include potential issues with data quality and completeness, selection bias due to the specific population or institution sampled, challenges in adequately accounting for confounding factors, variations in the timing of hematological parameter measurements, inability to establish causal relationships, limited generalizability beyond the studied ED, and the presence of potential biases and confounders. To obtain a comprehensive understanding of the limitations of this study, it is crucial to refer to the original publication and consider the details of the study design, sample size, data collection procedures, and any additional limitations acknowledged by the authors. This study on hematological parameters in COVID-19 patients has strengths in its retrospective design, ethical approval, comprehensive analysis of multiple parameters, appropriate statistical tests, and practical implications for clinical practice.

## Conclusions

The findings of this retrospective evaluation have the potential to contribute significantly to the understanding of COVID-19 pathophysiology and disease management. Identifying specific hematological parameters that correlate with disease severity and outcomes can assist healthcare professionals in making informed decisions regarding patient triage, resource allocation, and implementation of targeted interventions. By examining a large cohort of patients, we sought to elucidate the potential clinical significance of these parameters in COVID-19 and their association with disease severity and outcome. The results of this study have the potential to enhance our understanding of COVID-19 pathophysiology and aid in the development of effective management strategies to combat the ongoing global health crisis.
